# New insights into mechanisms of enhanced synaesthetic memory: Benefits are synaesthesia-type-specific

**DOI:** 10.1371/journal.pone.0203055

**Published:** 2018-09-05

**Authors:** Katrin Lunke, Beat Meier

**Affiliations:** Institute of Psychology, University of Bern, Bern, Switzerland; University of Zurich, SWITZERLAND

## Abstract

The goal of this study was to test the nature of the memory advantage in synaesthesia. We compared four different types of synaesthetes (27 grapheme-colour, 21 sound-colour-, 25 grapheme-colour-and-sound-colour- and 24 sequence-space synaesthetes) to their matched controls. Recognition memory for three types of stimuli (music, words, colour) was tested. We anticipated a general advantage in memory for synaesthetes and potentially additional synaesthesia-specific benefits. The results showed a general advantage for synaesthesia. Further, a benefit for colour stimuli resulted for grapheme-colour synaesthetes and a benefit for music stimuli resulted for grapheme-colour-and-sound-colour synaesthetes, indicating synaesthesia-type specific effects. These results suggest different mechanisms for the explanation of the memory benefit for different types of synaesthesia such as synaesthesia-related colour expertise for grapheme-colour synaesthesia and additional encoding opportunities for grapheme-colour-and-sound-colour synaesthesia.

## Introduction

Synaesthesia is a rare phenomenon in which the perception of ordinary stimuli (referred to as inducers) triggers untypical experiences (referred to as concurrents). Many different types of inducer-concurrent pairings exist. For example, in grapheme‐colour synaesthesia a digit (e.g., “5”) may trigger a specific colour experience (e.g., “blue). In sound-colour synaesthesia, music, tones and sounds trigger colour experiences. In sequence-space synaesthesia, sequences like days of the week or months elicit visuospatial representations. In case- and group studies grapheme-colour synaesthetes have shown cognitive benefits in many memory tasks (for reviews see [1, 2]).

However, only very few studies have considered memory in other types of synaesthesia, and no study so far has used inducer- and concurrent-specific as well as synaesthesia-unrelated material [3],[4]. The goal of the present study was to systematically compare different types of synaesthesia with different inducer-concurrent pairings regarding a general, an inducer- and a concurrent-related advantage in memory. Theoretically, several mechanisms might lead to a synaesthetic memory advantage and these might differ in different types of synaesthesia. A memory advantage for material related to the inducer would be consistent with dual‐coding theory as the concurrent experience leads to an encoding via two pathways [5]. Another possible explanation is the experience-driven expertise for materials of the synaesthetic domain that should be reflected in a memory advantage for stimuli related to inducer and/or concurrent. A third possibility is a general memory advantage through a broader and more connected semantic network in which information can be easily integrated [2],[6]. In the following we outline these three possible mechanisms and the evidence supporting each.

### Inducer-specific advantage

According to dual-coding theory the opportunity to encode a stimulus via two pathways increases the chances of remembering it compared to a stimulus that was coded only via one pathway [5]. The additional memory code (i.e., the synaesthetic concurrent) results in a stronger representation, additional retrieval cues, and accordingly, in a performance advantage for synaesthetes compared to non‐synaesthetes. Naturally, different types of synaesthesia would have benefits in different tasks according to their inducer. Several group studies do support the hypothesis of an inducer-specific benefit. Simner, Mayo and Spiller [3] found superior memory for inducer-related autobiographical and public dates in time-space synaesthetes. Radvansky, Gibson and McNerney [7] found superior memory performance for inducer-related words in grapheme-colour synaesthesia. In contrast, Rothen and Meier [8] did not find an inducer-related memory advantage in grapheme-colour synaesthetes in a free recall test of digit matrices. As, these studies did exclusively test memory for inducer-specific material in one single type of synaesthesia, they cannot inform whether potential benefits would occur for types of synaesthesia with different inducers.

### Domain-specific advantage

Another possible explanation for an advantage in memory for both inducer- and concurrent-related material is the specific expertise for the synaesthetic domain. Synaesthetes might benefit from easier encoding and processing of synaesthesia-related stimuli such as colours, or more generally, visually presented stimuli [6], [4], [9]. Additionally, according to the implicit bi-directionality of synaesthesia, an association also exists between the concurrent and the inducer. This association might provide additional retrieval cues on an implicit level for concurrent-related stimuli as well [10], [11], [12], [13],[6], [14]. Such a domain-specific account is in line with results by Yaro and Ward [9], who found that grapheme-colour synaesthetes outperformed their control participants in the inducer-related Rey auditory-verbal learning task and in concurrent-related colour perception and colour recognition tasks.

### General advantage

Neither dual-coding, implicit bi‐directionality, nor domain-expertise can account for a benefit for materials completely unrelated to synaesthesia [15],[16],[17]. It is possible, that the richer world of experiences with cascadically triggered associations in a broader semantic network accounts for this seemingly independent benefit, cf. [18], [19]. Such an explanation would be consistent with evidence from structural brain imaging suggesting a different, hyper‐connected network‐organization [20] and complemented by findings of general differences in cognitive style [21]. Accordingly, the memory advantage in synaesthesia would not necessarily directly be related to inducer and concurrent but rather to wider changes in the synaesthetic brain, related to perception and encoding in general [22], [23], [6]. This theory is in line with several group studies. Rothen and Meier [17] for instance found that grapheme-colour synaesthetes outperformed their control participants in inducer- and concurrent-related as well as synaesthesia-unrelated tasks of the Wechsler Memory Scale. Gross, Neargarder, Caldwell-Harris and Cronin-Golomb[24] on the other hand found an advantage for grapheme-colour synaesthetes only for a part of the inducer-related verbal and synaesthesia-unrelated tasks. These results rather support a general benefit but indicate that a benefit may only occur under certain circumstances. Pritchard, Rothen, Coolbear and Ward [16] tested recognition memory for shape-colour-location combinations. Grapheme-colour synaesthetes outperformed their control participants overall, strongest though when colour was the critical feature. This supports a concurrent expertise explanation. Bankieris and Aslin [15] compared grapheme-colour synaesthetes performance in learning colour-shape pairings. Synaesthetes outperformed controls which supports a more general memory advantage. However, neither Pritchard et al. [16] nor Bankieris and Aslin [15] included any inducer-related tasks.

Finally, Ward, Hovard, Jones and Rothen [4] used inducer-related, concurrent-related, and synaesthesia-unrelated memory tasks, based on words, non-words, scenes and abstract pictures (with and without colours), to compare grapheme-colour and lexical-gustatory synaesthetes. Grapheme-colour synaesthetes outperformed controls in all tasks, strongest for non-coloured fractals. Lexical-gustatory synaesthetes did not show any benefit. This speaks strongly against an inducer-specific benefit and supports a general advantage, but only for grapheme-colour synaesthetes. Further, it supports a mechanism related to coloured concurrents that appears to boost visual perception. Most importantly, it suggests that different types of synaesthesia have different memory advantages. This is consistent with the observation that different types of synaesthetes show preferences for different cognitive styles [21].

To summarize, the reviewed results support a memory advantage for grapheme-colour and for time-space synaesthesia [3], [4]. However, they also implicate different benefits for different types of synaesthesia. At this point, no conclusion can be drawn which type of synaesthesia benefits from which of the aforementioned mechanisms–inducer-, concurrent- or domain-specificity or a general advantage. First of all, there is evidence that types of synaesthesia differ. Moreover, it is likely that types tested as grapheme-colour synaesthetes so far have been heterogeneous as well. While some studies did not describe their sample further, other authors included grapheme-colour synaesthetes with colour experiences for auditory graphemes or such with colour experiences for printed graphemes. Still others tested synaesthetes with colour experiences for sequential words like months [25], [26],[7], [9]. Some synaesthetes do associate their visual concurrents like colours or spatial arrangements and some do project them into space [27]. Additionally, many synaesthetes do not only show one distinct type of synaesthesia but also other, partly less pronounced types. Synaesthetes with coloured concurrents show for instance a significantly higher prevalence for sequence-space synaesthesia [28], [29]. It is thus particularly difficult to create homogenous and distinctive groups of synaesthesia types to detect the possible source of an advantage through the material.

**Fig 1:**
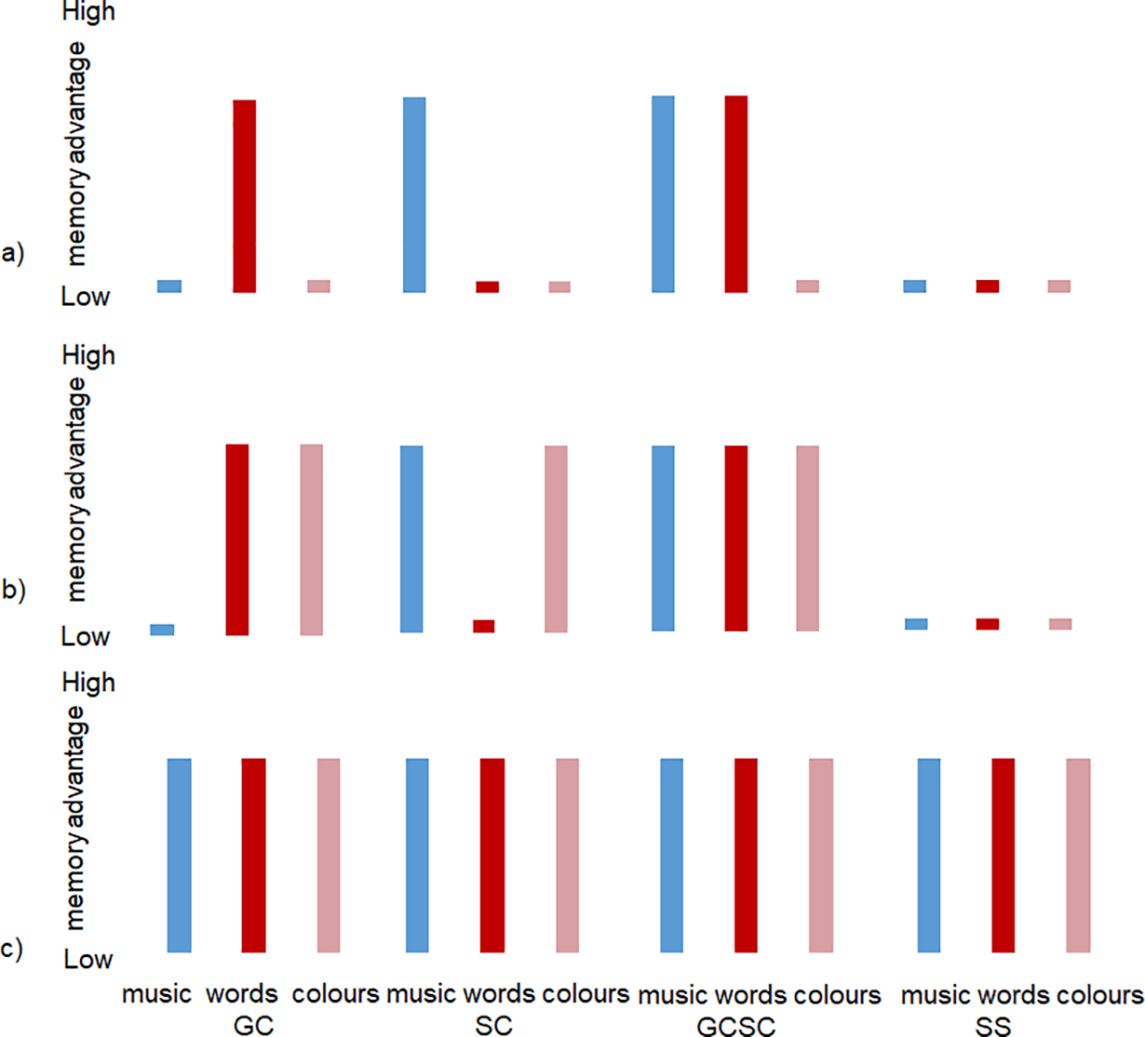
Expected results for the three possible mechanisms as mean differences between synaesthetic performance and performance of matched controls: a) an inducer-specific, b) a domain-specific and c) a general memory advantage of synaesthetes. GC = Grapheme-colour synaesthetes; SC = Sound-colour synaesthetes; GCSC = Grapheme-and-Sound-colour synaesthetes; SS = Sequence-Space synaesthetes.

Second, so far no study has included inducer-specific, concurrent-specific and unrelated material in a comparable way for different types of synaesthesia. In the present study, we thus compared four types of synaesthesia and matched controls for inducer-related, concurrent-related and synaesthesia-unrelated material in a recognition memory task. Grapheme-colour synaesthetes can be considered as the standard type of synaesthesia, sound-colour synaesthetes are based on a different inducer and grapheme-colour-and-sound-colour synaesthetes represent a multiple type of synaesthesia with different modalities. Additionally, we included sequence-space synaesthetes who have neither graphemes nor sound as inducer, but sequences, and no coloured concurrents, but spatial representations. All participants completed three episodic recognition memory tests, that is, one for words, one for music, and one for colour stimuli. Fig 1 illustrates the extent of the expected advantage compared to matched controls according to the three theories described above. As evidence for an inducer-specific advantage we expected a higher performance for grapheme-colour and grapheme-colour-and-sound-colour synaesthetes regarding word stimuli and for sound-colour synaesthetes and grapheme-colour-and-sound-colour synaesthetes regarding musical stimuli. As evidence for a domain-specific advantage we expected a higher performance for grapheme-colour-, sound-colour- and grapheme-colour-and-sound-colour synaesthetes regarding coloured stimuli and for their inducing stimuli respectively. If synaesthesia would show a general memory advantage, we expected higher performance in all types of stimuli.

## Method

### Participants

We recruited 102 synaesthetes who had completed the Synaesthesia-Check on our website (www.synaesthesie.unibe.ch) previously, and who had reported consistent colours for words, digits, letters, musical tones, instruments or melodies and/or spatial representations for digits, days, months or years. 102 healthy control-participants matched for age, gender and education were recruited, cf. [30]. The study was approved by the Ethics committee of the Human Sciences Faculty of the University of Bern (#2013-1-272903). Five participants experienced technical problems during different tasks and were therefore eliminated together with their matched controls resulting in a sample size of 97 synaesthetes and 97 control participants. Of the synaesthetes, 27 were grapheme-colour synaesthetes (25 female and 2 male), 21 were sound-colour- (14 female and 7 male), 25 were grapheme-colour-and-sound-colour (21 female and 4 male) and 24 sequence-space synaesthetes (23 female and 1 male). Nine synaesthetes and nine controls were left handed. Table 1 shows the demographic characteristic of all groups.

**Table 1:** Age, education and verbal intelligence of each type of synaesthesia and the respective control group.

		Synaesthetes	Controls
		*M* (*SD*)	*M* (*SD*)
	age	42.22 (17.81)	42.78 (18.56)
GC	years of primary education	11.93 (1.54)	12.07 (1.85)
	verbal IQ	111.04 (9.09)	107.82 (11.12)
	age	36.43 (19.11)	37.38 (20.16)
SC	years of primary education	12.48 (1.21)	12.95 (1.72)
	verbal IQ	111.76 (10.07)	109.90 (9.54)
	age	32.44 (13.34)	32.80 (13.29)
GCSC	years of primary education	11.46 (1.68)	12.54 (1.54)
	verbal IQ	106.92 (6.56)	107.52 (7.54)
	age	34.46 (15.56)	34.46 (15.97)
SS	years of primary education	12.71 (1.30)	12.06 (1.34)
	verbal IQ	113.17 (9.09)	106.96 (9.71)

*Note*. GC = Grapheme-colour synaesthetes; SC = Sound-colour synaesthetes; GCSC = Grapheme-and-Sound-colour synaesthetes; SS = Sequence-Space synaesthetes. Verbal IQ was tested with a standardized vocabulary test [31].

One-factorial ANOVAs showed no difference between synaesthetes and controls for age, *F*(1, 192) = 0.04, *MSE* = 288.90, *p* = .853, η_p_^2^ < .01 and years of primary education *F*(1, 192) = 1.34, *MSE* = 2.50, *p* = .248, η_p_^2^ < .01. However, there was an effect of Verbal IQ which was due to sequence-space synaesthetes who showed a higher score than their matched controls (*t*(46) = 2.29, *SE* = 2.72, *p* = .014).

Prior to participation in the laboratory, participants with colour experiences for graphemes filled out an on-line measurement of consistency. They were presented with 36 black on white graphemes (A-Z, 0–9) in a random order and instructed to choose a colour out of 13 (black, dark blue, brown, dark green, grey, pink, purple orange, red, white, light blue, light green, yellow) or indicate *no colour* [32], [29]. The mean number of consistent digits and letters was *M* = 25.19 (*SD* = 6.97) which is above the cut-off of 20 used by Simner et al. [29] and Rothen and Meier [32]. Two participants did not fill out the on-line measurement. Six of the 52 participants with grapheme-colour synaesthesia scored lower than 20 consistent digits and letters. Notably, three of the remaining six participants had only digit-colour or word-colour synaesthesia. However, we did not exclude any participant as we relied on the subjective experiences of the participants. Notably, a re-analysis without these three participants gave the same pattern of results.

For sound-colour synaesthesia, we originally considered using the Eagleman battery [33]. However, we noticed that in this battery, using a simple strategy (low tones/big instrument -> dark colours; high tones/small instruments -> bright colours) leads to a high consistency score (passing synaesthesia criterion). Moreover, sound-colour synaesthesia is a very heterogeneous phenomenon in which for some people pitch relates to colours, for others timbre relates to colours, for others tone intervals relate to colours, etc.. Thus, we decided to rely on the subjective self-reported experiences.

For sequence space synaesthesia, participants who experienced spatial representations for sequences were instructed to draw these representations once at the beginning and once at the end of the experiment on-site (about 2h later). Drawings were categorized by an independent rater regarding consistency and complexity to confirm synaesthesia. One synaesthete was rated as inconsistent. However, this participant had indicated where variations occur in her experiences and was consistent in some other. We thus did not exclude her.

At the beginning of the laboratory session, both synaesthetes and control participants were asked again whether they experienced any kind of synaesthesia. If any synaesthetic experiences were reported in addition to those described in the questionnaire, participants were–if possible–tested for consistency and either reassigned or excluded. If several types of synaesthesia were present, participants were asked which they would describe as the main type of synaesthesia. Of the grapheme-colour synaesthetes, thirteen reported having additional types of synaesthesia (sequence-space, other: person-smell and situation-smell). One grapheme-colour synaesthete was recategorised as grapheme-colour-and-sound-colour synaesthete. One control participant was recategorised as grapheme-colour synaesthete. Of the sound-colour synaesthetes, thirteen reported additional types of synaesthesia (sequence-space, grapheme-colour, other: ticker-tape, touch-colour, smell-form, two person/memory-colour, taste-colour/pictures). Three grapheme-colour-and-sound-colour synaesthetes were recategorised as sound-colour synaesthetes. Moreover, one control participant was recategorised as sound-colour synaesthete. Of the grapheme-colour-and-sound-colour synaesthetes, fourteen reported additional types of synaesthesia in the laboratory (sequence-space, other: ticker-tape, daytime-feeling, pain-colour, scene-taste/smell, sound-haptic). Three grapheme-colour synaesthetes were recategorised as grapheme-colour-and-sound-colour synaesthetes. Of the sequence-space synaesthetes, three reported additional types of synaesthesia in the laboratory (one grapheme-colour, one grapheme-colour-and-sound-colour, one person/experience-colour). One grapheme-colour and one grapheme-colour-and-sound-colour synaesthete were recategorised as sequence-space synaesthetes. Four control participants were recategorised as sequence-space synaesthetes.

To obtain a medium effect size as reported in previous research, e.g. [17], for a repeated measures ANOVA with a three-levels within-subjects factor and 1-β = .80, the G*Power analysis proposed 19.13 participants per group [34]. We aspired to test at least 20 participants per group.

### Material

#### Word recognition

The word material was composed according to an earlier study [35] and consisted of high‐frequency and low‐frequency words, all concrete nouns selected from the vocabulary database of the University of Leipzig (http://wortschatz.uni-leipzig.de/). Two counter-balanced study and test lists were constructed. Twenty-four words of each frequency category were used in the study list together with 19 filler words (days and months), the remaining 24 words of each category were used as lures in the recognition test. Mean word-length was 5.42 letters. In the study phase, words were presented in a 32 point black Arial font on a white rectangle. A chart of colours was presented with every word. Each consisted of the 10 colours red, navy, yellow, green, black, white, light blue, purple, pink and brown, illustrated as 2x1.2cm big squares, arranged in an upright rectangle. In the recognition phase, words were presented in an 18 point courier new font centred on a white background.

#### Music recognition

Each piece of music was selected from unfamiliar, rare recordings. They comprised music styles such as Classic, Jazz, Rock, Pop, Metal, Chinese, Indian, and Swiss folklore. Ten sec wav‐files were cut. Two counter-balanced study and test lists were constructed. Twenty-four pieces were used in the study list, 24 additional pieces were used as lures in the recognition test. Loudness was set to a comfortable level for the participants.

#### Colour recognition

Colour patterns were selected for colour recognition. Half were selected from a synaesthesia catalogue (International Congress on Synesthesia, Science and Art, Granada, 2009) and half of the patterns were Mondrian style pictures, each consisting of four differently coloured squares. Two counter-balanced study and test lists were constructed. Twenty-four patterns (twelve of each category) were used in the study list, 24 patterns (twelve of each category) were used as lures in the recognition test. The pictures were presented centred on a white screen (50% height x 50% width) in the study as well as the recognition phase.

#### Verbal intelligence

To assess verbal intelligence, a standardized vocabulary test was used. It consists of 42 trials, each composed of one target word and five distractor pseudo-words and the participant has to select the real world [31].

### Apparatus

On-site, participants were tested under controlled light conditions with an 85lux/watt lamp with 6400 calvin colour temperature and two standard interior lamps. Stimuli were presented with E-prime 1.2 (https://www.pstnet.com) on a standard 17 inch flat screen. Answers were given on a standard keyboard and sound was delivered via standard Sennheiser stereo headphones. Audio output was set at a comfortable level for the participant and remained unchanged during data collection. For one participant headphones were replaced by speakers due to her sensitivity of the ears.

### Procedure

Participants received a brief information about synaesthesia and signed a consent form. In the course of the acquisition of demographic data, synaesthetes who experience spatial representations for sequences sketched their representations.

In the study phase, participants were first presented with a list of words on the screen one at the time. In addition to each word, a colour palette with 13 different colours was presented and the participant was instructed to select the colour that goes best with the particular word. Words were presented in randomized order for each participant. After a colour was selected the next word appeared immediately. For the music study phase, participants were asked to put on headphones. They were presented with short pieces of music for 10 sec each and were instructed to rate on a seven‐point scale how much they liked it. They were also asked whether they knew this particular piece of music immediately after each stimulus. Musical pieces were presented in randomized order for each participant. For the colour study phase, participants were presented with coloured patterns for 3 seconds each and they were asked to rate how much they liked each pattern on a seven-point Likert scale. Colour patterns were presented in randomized order for each participant. The three study phases were always fulfilled in the same order: words–music–pictures.

After a filled retention interval of approximately 60 minutes, including the vocabulary test [31], the memory test phase began. In the word recognition test phase, words were presented, one at the time, in randomized order for each participant at the center of the screen, in black on a white background. Participants were informed that some of the words were old words from the study phase and some were new words not presented before. They were instructed to indicate whether the word was old or new. After a “new” decision, the next word appeared immediately. An “old” decision was given by pressing “b” and a “new” decision by pressing “n”. After an “old” decision, participants were immediately asked to give a Remember/Know judgement. They were instructed to give a “remember” response when they were able to recollect the word from the study phase and to give a “know” response when they were not able to recollect the word, but nevertheless believed that they had seen it in the study phase. They were instructed to press key 1 after each response they had felt they exactly remembered the stimulus and key 2 whenever they had the feeling they remembered the stimulus. After a response was made, the next word appeared.

In the music recognition test phase, participants were again asked to put on headphones. They were played pieces of music for 10 sec each and they were informed that some of the pieces had been played before in the study phase (old pieces) and some not (new pieces). The pieces were presented in randomized order for each participant. They were instructed to indicate whether a piece was old or new. Immediately after an “old” decision, participants were asked to give a Remember/Know judgement similar as in the word-recognition phase.

In the colour recognition test, coloured patterns were presented, one at the time, in randomized order at the center of the screen. Participants were informed that some of the patterns were old patterns from the study phase and some were new patterns not presented before. Colour patterns were presented in randomized order for each participant. They were instructed to indicate whether a pattern was old or new. Immediately after an “old” decision, participants were asked to give a Remember/Know judgement as in the word-recognition phase.

After the three tests were completed, sequence-space synaesthetes were asked to draw their sequences for a second time.

### Statistical analysis

Alpha was set at .05 for all analyses. Analyses were primarily performed in SPSS [36]. If homogeneity of variances was violated Greenhouse-Geisser corrected values are reported. In order to be able to interpret non-significant effects between synaesthetes and controls, Bayesian statistics was calculated using JASP [37].

Between-subject factors were *synaesthesia* (yes/no) with two levels and *type of synaesthesia* (grapheme-colour, sound-colour, grapheme-colour-and-sound-colour, sequence-space) with four levels. Within-subjects factor was *type of stimuli* (words, music, colours) with three levels. *Age* differed between *types of synaesthesia* and was thus included as a covariate.

To assess recognition for words, music and colours, hits and false alarms were assessed and the proportions of recognition (Pr) were computed by individually subtracting false alarms from hits. This score reflects the two-high-threshold model of recognition in which old items are correctly categorised as old when they exceed the recognition threshold and new items are wrongly categorised as old when they exceed the recognition threshold [38]. For an additional visualisation of performance differences between synaesthetes and non-synaesthetes, we z-transformed Pr scores for each type of synaesthesia and their controls separately and then subtracted scores of controls from scores of synaesthetes in order to provide a depiction that is comparable to the expected results in Fig 1.

As measurement of response bias, *C* was computed as -0.5 * (zHits + zFalseAlarms) [39]. Remember/Know judgements were analysed as estimates of recollection and familiarity according to the formula: Recollection = [(Remember old − Remember new)/(1 − Remember new)]; Familiarity = [z(Familiarity old) − z(Familiarity new)], with Familiarity old = [Know old/(1 − Remember old)] and Familiarity new = [Know new/(1 − Remember new)] by Yonelinas, Kroll, Dobbins, Lazzara and Knight [40]. Extreme values in hits and false alarms (e.g. 0, 24, 48) were adjusted by adding 0.5 to values of 0 and substracting 0.5 from values of 24 or 48 respectively before the proportion was calculated [41].

## Results

### General memory performance

**Fig 2:**
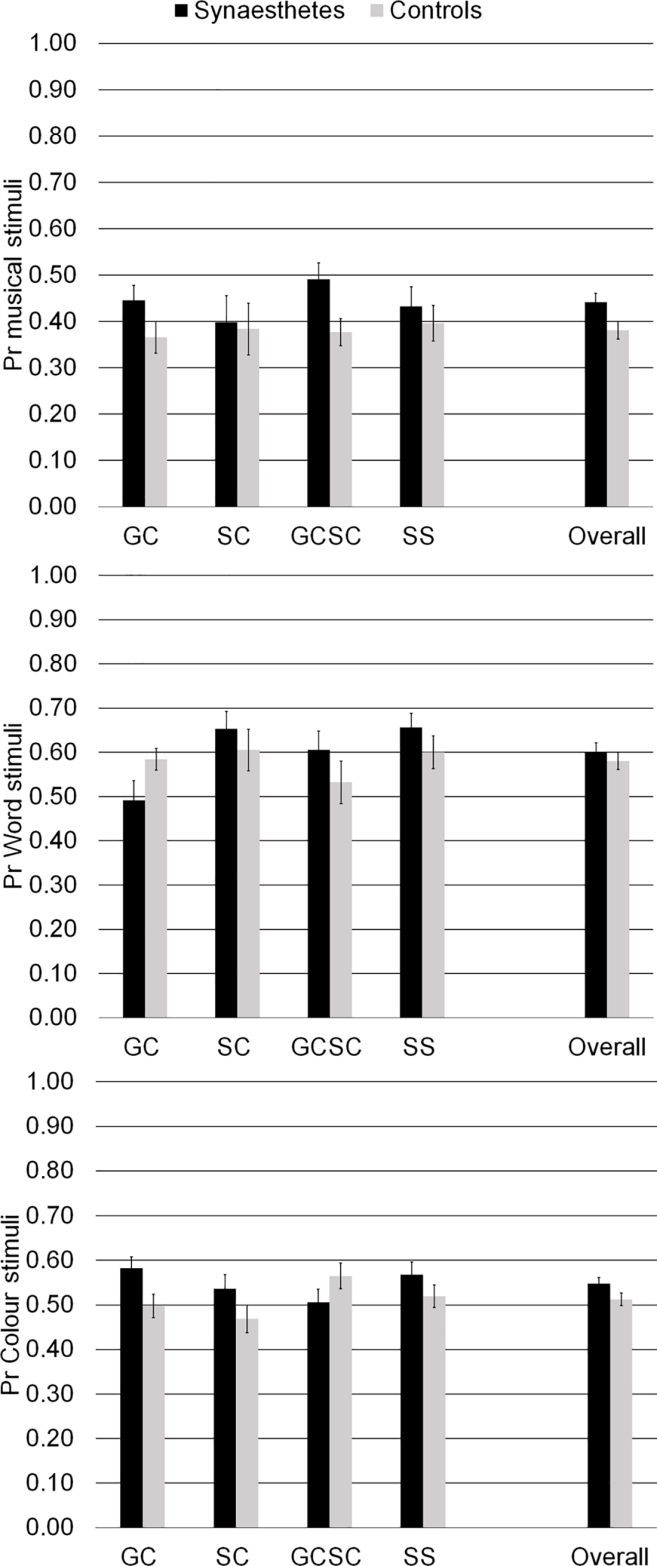
Recognition memory performance (Pr) for musical stimuli, word stimuli and colour stimuli. GC = Grapheme-colour synaesthetes; SC = Sound-colour synaesthetes; GCSC = Grapheme-and-Sound-colour synaesthetes; SS = Sequence-Space synaesthetes. Error bars display standard errors.

The results are presented in Fig 2. We conducted a 2x4x3 repeated measures ANCOVA with the between-subject factors *synaesthesia* (yes/no), *type of synaesthesia* and the within-subjects factor *type of stimuli*. *Age* was included as covariate. Dependent variable was memory performance calculated as Pr. A significant main effect of *synaesthesia* (yes/no) *F*(1, 185) = 5.87, *MSE* = .03, *p* = .016, η_p_^2^ = .03 occurred as well as a two-way interaction between *type of synaesthesia* and *type of stimuli*, *F*(5.66, 349.28) = 2.21, *MSE* = .03, *p* = .046, η_p_^2^ = .04 and a three-way interaction between *synaesthesia* (yes/no), *type of synaesthesia* and *type of stimuli F*(5.66, 349.28) = 3.14, *MSE* = .03, *p* = .006, η_p_^2^ = .05. The covariate *age* produced a main effect, *F*(1, 185) = 65.29, *MSE* = .03, *p* < .001, η_p_^2^ = .26 and interacted with *type of stimuli*, *F*(1.89, 349.28) = 6.17, *MSE* = .03, *p* = .003, η_p_^2^ = .03. We addressed the three-way interaction between *synaesthesia* (yes/no), *type of synaesthesia* and *type of stimuli* by separating the analysis for *type of synaesthesia* which also allowed us to see for synaesthesia specific differences in stimuli that act as inducer or concurrent. Four separate ANCOVAs including *synaesthesia* (yes/no) as between-subject factor, *type of stimuli* as within-subject factor and *age* as covariate were conducted.

**Fig 3:**
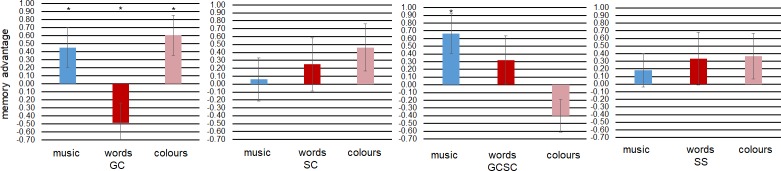
Difference of z-transformed recognition (Pr) of synaesthetes and controls. Sound-colour- and sequence-space synaesthetes show a general advantage. Grapheme-colour-and-sound-colour synaesthetes show an inducer-specific advantage. Grapheme-colour synaesthetes show rather a concurrent-related advantage. GC = Grapheme-colour synaesthetes; SC = Sound-colour synaesthetes; GCSC = Grapheme-and-Sound-colour synaesthetes; SS = Sequence-Space synaesthetes. Error bars display standard errors.

In order to specifically illustrate the effects of synaesthesia, we plotted the difference of z-transformed on recognition memory performance in Fig 3 to provide a direct opportunity for visual comparison for the theoretical predictions outlined in Fig 1. An analysis including intelligence as a covariate is provided in the supporting information.

### Grapheme-colour synaesthetes

No main effect resulted for *synaesthesia* (yes/no), *F*(1, 51) = 0.60, *MSE* = .03, *p* = .441, η_p_^2^ = .01. An interaction occurred between *synaesthesia* (yes/no) and *type of stimuli F*(2, 102) = 6.81, *MSE* = .02, *p* = .002, η_p_^2^ = .12. The covariate *age* produced a main effect, *F*(1, 51) = 18.08, *MSE* = .03, *p* < .001, η_p_^2^ = .26 but did not interact. Post-hoc tests showed that grapheme-colour synaesthetes had an advantage for colour stimuli, *t*(52) = 2.32, *SE* = .04, *p* = .012, *d* = 0.63 and for music, *t*(52) = 1.69, *SE* = .05, *p* = .049, *d* = 0.46 and a disadvantage for words, *t*(40.29) = -1.85, *SE* = .05, *p* = .036, *d* = -0.50.

### Sound-colour synaesthetes

No main effect resulted for *synaesthesia* (yes/no), *F*(1, 39) = 1.22, *MSE* = .04, *p* = .276, η_p_^2^ = .03 and no interaction between *synaesthesia* (yes/no) and *type of stimuli F*(2, 78) = 0.32, *MSE* = .03, *p* = .726, η_p_^2^ = .01. The covariate *age* produced a main effect, *F*(1, 39) = 21.18, *MSE* = .04, *p* < .001, η_p_^2^ = .35 but did not interact. Bayesian *t*-tests confirmed these results by showing evidence for the null hypothesis for musical stimuli, *BF*_10_ = 0.31. For colour stimuli, *BF*_10_ = 0.75 and word stimuli, *BF*_10_ = 0.39 results were inconclusive.

### Grapheme-colour-and-sound-colour synaesthetes

No main effect resulted for *synaesthesia* (yes/no), *F*(1, 47) = 1.74, *MSE* = .04, *p* = .194, η_p_^2^ = .04. An interaction occurred between *synaesthesia* (yes/no) and *type of stimuli F*(1.68, 78.70) = 3.70, *MSE* = .03, *p* = .036, η_p_^2^ = .07. The covariate *age* produced a main effect, *F*(1, 47) = 12.59, *MSE* = .04, *p* = .001, η_p_^2^ = .21 but did not interact. Post-hoc tests showed that grapheme-colour-and-sound-colour synaesthetes had a significant advantage for music stimuli, *t*(48) = 2.48, *SE* = .05, *p* = .009, *d* = 0.70 but not for words, *t*(48) = 1.14, *SE* = .07, *p* = .131, *d* = 0.32 or colours, *t*(48) = -1.41, *SE* = .04, *p* = .082, *d* = -0.40. Results of Bayesian *t*-tests regarding colour stimuli, *BF*_10_ = 0.64 and word stimuli, *BF*_10_ = 0.48 were inconclusive.

### Sequence-space synaesthetes

No main effect resulted for *synaesthesia* (yes/no), *F*(1, 45) = 2.51, *MSE* = .03, *p* = .120, η_p_^2^ = .05 and no interaction between *synaesthesia* (yes/no) and *type of stimuli F*(2, 90) = 0.06, *MSE* = .02, *p* = .938, η_p_^2^ < .01. The covariate *age* produced a main effect, *F*(1, 45) = 12.47, *MSE* = .03, *p* = .001, η_p_^2^ = .22 and interacted with *type of stimuli*, *F*(2, 90) = 11.30, *MSE* = .02, *p* < .001, η_p_^2^ = .20. Bayesian *t*-tests gave inconclusive results for colour stimuli, *BF*_10_ = 0.56, for word stimuli, *BF*_10_ = 0.50 and for musical stimuli, *BF*_10_ = 0.34.

### Response bias

To assess whether synaesthetes differ in their general tendency to respond yes, response bias was computed as *C* and included as dependent variable in a mixed ANCOVA with between-subjects factors *synaesthesia* (yes/no) and *type of synaesthesia* [39]. The within-subjects factor was *type of stimuli* and the covariate *age*. No main effect occurred for *synaesthesia* (yes/no), *F*(1, 185) = 0.07, *MSE* = .92, *p* = .792, η_p_^2^ < .01 and *type of synaesthesia*, *F*(3, 185) = 0.31, *MSE* = .92, *p* = .819, η_p_^2^ < .01 and no interaction between the two, *F*(3, 185) = 1.07, *MSE* = .92, *p* = .362, η_p_^2^ = .02 or between *synaesthesia* (yes/no) and *type of synaesthesia* and *type of stimuli*, *F*(6, 370) = 0.15, *MSE* = .44, *p* = .990, η_p_^2^ < .01. A main effect occurred for *type of stimuli*, *F*(2, 370) = 11.29, *MSE* = .44, *p* < .001, η_p_^2^ = .06. *Type of stimuli* interacted with the covariate *age*, *F*(2, 370) = 13.71, *MSE* = .44, *p* < .001, η_p_^2^ = .07. Bayesian *t-*tests gave evidence for the null hypothesis for grapheme-colour synaesthetes regarding all types of stimuli, all *BF*_10_ between 0.27 and 0.32 and for sound-colour synaesthetes regarding music, *BF*_10_ = 0.33 and colours, *BF*_10_ = 0.31. All other results were inconclusive, all *BF*_10_ between 0.34 and 0.67.

### Recollection and familiarity

To assess whether synaesthetes differ in retrieval strength we analysed recollection and familiarity as suggested by Yonelinas et al. [40]. We conducted two separate repeated measures ANCOVAs–one for recollection and one for familiarity–with between-subject factors *synaesthesia* (yes/no) and *type of synaesthesia* (Table 2). The within-subjects factor was *type of stimuli* and the covariate *age*. For estimates of recollection, a main effect occurred for *synaesthesia* (yes/no), *F*(1, 185) = 5.41, *MSE* = .05, *p* = .021, η_p_^2^ = .03 but not for *type of synaesthesia*, *F*(3, 185) = 0.44, *MSE* = .05, *p* = .725, η_p_^2^ < .01 and no interaction between the two, *F*(3, 185) = 1.53, *MSE* = .05, *p* = .207, η_p_^2^ = .02. An interaction occurred between *type of synaesthesia* and *type of stimuli*, *F*(5.23, 322.73) = 2.92, *MSE* = .03, *p* = .012, η_p_^2^ < = .05. The covariate *age*, produced a main effect, *F*(1, 185) = 21.74, *MSE* = .05, *p* < .001, η_p_^2^ = .11 and interacted with *type of stimuli*, *F*(1.74, 322.72) = 5.99, *MSE* = .03, *p* = .004, η_p_^2^ = .03. Post-hoc tests showed for grapheme-colour synaesthetes significantly higher recollection for colours, *t*(52) = 1.74, *SE* = .04, *p* = .044, *d* = 0.47 and lower recollection for words, *t*(43.26 = -1.81, *SE* = .06, *p* = .04, *d* = -0.49. For sound-colour synaesthetes they showed a significantly higher recollection for colour stimuli, *t*(40) = 2.40, *SE* = .05, *p* = .011, *d* = 0.74 and music, *t*(40) = 2.44, *SE* = .06, *p* = .010, *d* = 0.75. Bayesian *t-*tests showed that for grapheme-colour synaesthetes the results for music were inconclusive, *BF*_10_ = 0.35. For sound-colour synaesthetes the result for words was inconclusive, *BF*_10_ = 0.51. For grapheme-colour-and-sound-colour synaesthetes evidence for the null hypothesis occurred regarding words, *BF*_10_ = 0.28 and colours, *BF*_10_ = 0.30. Results regarding music were inconclusive, *BF*_10_ = 0.52. For sequence-space synaesthetes results were inconclusive, all *BF*_10_ between 0.40 and 0.47.

**Table 2:** Estimates of recollection and familiarity for each type of synaesthesia and stimuli.

		GC	Controls	SC	Controls	GCSC	Controls	*SS*	Controls
		*M* (*SD*)	*M* (*SD*)	*M* (*SD*)	*M* (*SD*)	*M* (*SD*)	*M* (*SD*)	*M* (*SD*)	*M* (*SD*)
music	recollection	0.33 (0.21)	0.29 (0.16)	0.38 (0.16)	0.24 (0.21)	0.40 (0.22)	0.32 (0.21)	0.33 (0.19)	0.28 (0.18)
	familiarity	0.36 (1.24)	-0.17 (1.16)	-0.26 (1.41)	-0.14 (1.78)	0.29 (1.15)	-0.35 (0.84)	0.11 (1.25)	0.07 (1.15)
words	recollection	0.47 (0.26)	0.58 (0.16)	0.69 (0.20)	0.63 (0.15)	0.58 (0.27)	0.58 (0.28)	0.68 (0.18)	0.62 (0.21)
	familiarity	-0.22 (1.21)	-0.10 (0.74)	0.16 (1.32)	-0.09 (1.25)	0.32 (0.93)	-0.16 (1.03)	0.13 (0.75)	0.00 (0.88)
colours	recollection	0.51 (0.16)	0.45 (0.13)	0.53 (0.18)	0.40 (0.15)	0.47 (0.17)	0.49 (0.17)	0.51 (0.14)	0.48 (0.15)
	familiarity	0.38 (1.07)	-0.12 (0.92)	-0.06 (1.11)	-0.37 (1.00)	-0.12 (1.16)	0.21 (0.97)	0.17 (1.00)	-0.19 (0.93)

*Note*. GC = Grapheme-colour synaesthetes; SC = Sound-colour synaesthetes; GCSC = Grapheme-and-Sound-colour synaesthetes; SS = Sequence-Space synaesthetes. Sound-colour- and sequence-space synaesthetes show a general advantage.

For familiarity, a main effect occurred for *synaesthesia* (yes/no), *F*(1, 185) = 4.28, *MSE* = 1.55, *p* = .040, η_p_^2^ = .02 and none for *type of synaesthesia*, *F*(3, 185) = 0.80, *MSE* = 1.55, *p* = .497, η_p_^2^ = .01 and no interaction between the two, *F*(3, 185) = 0.13, *MSE* = 1.55, *p* = .945, η_p_^2^ < .01 (Table 2). The covariate *age*, produced a main effect, *F*(1, 185) = 20.19, *MSE* = 1.55, *p* < .001, η_p_^2^ = .10 and interacted with *type of stimuli*, *F*(2, 370) = 7.30, *MSE* = .94, *p* = .001, η_p_^2^ = .04. Post-hoc *t*-tests showed a significantly higher familiarity for grapheme-colour synaesthetes regarding colours, *t*(52) = 1.87, *SE* = .27, *p* = .034, *d* = 0.51 and for grapheme-colour-and-sound-colour synaesthetes regarding music, *t*(43.79) = 2.24, *SE* = .28, *p* = .015, *d* = 0.63 and words, *t*(48) = 1.72, *SE* = .28, *p* = .047, *d* = 0.49. Bayesian *t-*tests showed for grapheme-colour synaesthetes evidence for the null hypothesis regarding words, *BF*_10_ = 0.30 and inconclusive results regarding music, *BF*_10_ = 0.80. For sound-colour synaesthesia there was evidence for the null hypothesis regarding music, *BF*_10_ = 0.31 and inconclusive results for words, *BF*_10_ = 0.35 and colours, *BF*_10_ = 0.44. For grapheme-colour-and-sound-colour synaesthetes there was inconclusive evidence for the effects of *synaesthesia* (yes/no) regardingr colours, *BF*_10_ = 0.46. For sequence-space synaesthetes there was evidence for the null hypothesis regarding musical stimuli, *BF*_10_ = 0.29 and words, *BF*_10_ = 0.32. Results were inconclusive regarding colours, *BF*_10_ = 0.58.

## Discussion

The goal of this study was to test the nature of the memory advantage in synaesthesia. Towards this goal, recognition memory performance of four different types of synaesthesia (grapheme-colour, sound-colour, grapheme-and-sound-colour, sequence-space) were compared to matched controls for three different types of stimuli, that is, inducer- or concurrent-specific or synaesthesia-unrelated. The results support an advantage for grapheme-colour synaesthetes for concurrent-related colour stimuli, also reflected in significantly higher recollection. In contrast, grapheme-colour-and-sound-colour synaesthetes showed a significant benefit for inducer specific sound-stimuli. These results suggest that different types of synaesthesia, regardless of overlapping inducer-concurrent pairings, rely on different mechanisms that support their specific memory advantage.

### General advantage

Across types of stimuli, synaesthetes showed higher recognition memory than the respective control groups. This general advantage was small but consistent for all types of synaesthesia. A general advantage would be in line with earlier research that found a memory advantage for synaesthesia-unrelated material, e.g. [24], [7], [17]. Overall synaesthetes did show significantly higher estimates of recollection based responses. This indicates richer memory traces and more retrieval strength for synaesthetes, [35], [42] and is in line with findings by Chin and Ward who found significantly more recollection for autobiographic memories in grapheme-colour synaesthetes [43]. These results support the hypothesis that not (only) the synaesthetic experience itself is boosting memory, but a more general cognitive difference expressed also in cognitive style and neural connectivity. Synaesthetes are supposed to have a broader, more connected semantic network. These differences are related to more efficient integration, propagation and processing of information. They are hypothesized to having a more associative semantic memory with richer representations and easier access. This accounts for an advantage beyond synaesthesia related material, [20], [2], [21], [44].

### Specific advantage

We also found an interaction between *synaesthesia* (yes/no), *type of synaesthesia* and *type of stimuli*, indicating an inducer-specific benefit for musical stimuli in grapheme-colour-and-sound-colour synaesthetes and a significant concurrent-specific benefit for colour stimuli in grapheme-colour synaesthetes. These results speak against a domain-specific or a general advantage. They are partly in line with earlier research that found differences between different types of synaesthesia [4]. However, we had rather expected consistent inducer- and/or concurrent-specific benefits for those types of synaesthetes who share a certain concurrent. Thus, if any, we anticipated the same specific benefits for grapheme-colour-, for sound-colour and for grapheme-colour-and-sound-colour synaesthetes, for instance, an advantage for visually presented word and colour stimuli. However, word stimuli led to the smallest and statistically non-significant benefit. Further, we had expected a possible colour-specific advantage would be present in all colour-synaesthetes, but only grapheme-colour synaesthetes showed a benefit. These are interesting findings as they indicate different mechanisms for different types of synaesthesia.

The enhanced recognition memory performance of grapheme-colour synaesthetes for colour stimuli occurred together with significantly higher recollection. This result may indicate that their memory traces were richer and retrieval strength was higher compared to their matched controls. Recollection is generally linked to learning of novel items and associations including context information [45], [46], [42]. According to models of recollection and familiarity it reflects storage in the episodic memory [42]. Thus, our results may imply that enhanced processing of colour stimuli leads to more efficient, controlled encoding, stronger memory traces in episodic memory and higher retrieval strength. This would be in line with findings by Pritchard et al. [16] who found a certain expertise for visual stimuli and with Yaro and Ward [9] and Rothen and Meier [17] who found better encoding and retention for visual stimuli. It would also be congruent with findings by Bankieris and Aslin, [15, 25] who found an enhanced ability to form stable colour associations.

The results show that significant advantages were only present for synaesthetes with grapheme-colour inducer-concurrent pairings and only for some synaesthesia-related stimuli. This is in line with Ward et al. [4] who found an advantage for grapheme-colour but not lexical-gustatory synaesthetes. That any other type of synaesthesia showed an advantage is partly in line with findings by Simner et al. [3] who found that sequence-space synaesthetes showed advantages only for stimuli related to their type of synaesthesia.

The difference between grapheme-colour-and-sound-colour synaesthetes and grapheme-colour synaesthetes may be due to the higher co-occurrence of several types of synaesthesia. Previous studies have revealed that subsamples with multiple types of synaesthesia differed from those with fewer or only one type. Meier and Rothen [21], for example, found that synaesthetes with multiple types differed in their cognitive style. Ward, Thompson-Lake, Ely and Kaminski [44] found that the number of types of synaesthesia correlated positively with convergent creative thinking. Especially the latter finding supports the view of a broader semantic network which is crucial for creative thinking and was also anticipated with inducer-specific memory benefits [6, 47].The inducer-specific advantage for musical stimuli in grapheme-colour-and-sound-colour synaesthetes could therefore imply that multiple types of synaesthesia do show the broader kind of semantic network hypothesized for synaesthetes in general [19]. However, this would suggest that monotypic types of synaesthesia or those for whom inducer and concurrent activate the same modality do not show this broader network activation but rely on other mechanisms such as enhanced colour processing. As stated in the participant section, a clear division between types of synaesthesia is very hard to obtain and our sample of monotypic synaesthetes contains participants who stated other, presumably less prominent types of synaesthesia when asked in the laboratory. However, in our sample, grapheme-colour-and-sound-colour synaesthetes are those with multiple synaesthetic experiences involving different modalities. In sum, these results support synaesthesia specific stronger memory advantages which are related to inducer or concurrent depending on the type of synaesthesia. Furthermore, the result that no type showed a significant advantage for words is in line with Teichmann, Nieuwenstein and Rich [14], who suggested that additional synaesthetic information only aids memory if it is less abstract than the stimulus to be learned.

Alike many other studies on synaesthesia, we cannot rule out a motivational difference between synaesthetes and controls. However, we used an incidental study phase to reduce this possibility. Moreover, the particular pattern of results does not suggest a motivational bias.

## Conclusion

Overall, this study shows a general memory advantage for different types of synaesthesia. An inducer-specific benefit was present for grapheme-colour-and-sound-colour synaesthetes, possibly through elevated activation in the semantic network. A concurrent-specific benefit was present for grapheme-colour synaesthetes, based on higher recollection, possibly related to enhanced expertise in colour processing. Interestingly, no evidence for a domain-specific benefit emerged in any condition.

The fact that the memory benefit of different types of synaesthesia is due to different mechanisms implicates strongly that it is important to investigate further types of synaesthesia in order to advance our understanding rather than generalize from one specific type. Moreover, it is also necessary to distinguish monotypic (e.g., sound-colour) from multiple types (e.g. grapheme-colour-and-sound-colour synaesthetes).

## Supporting information

S1 Table Proportions of hits and false alarms and C for each type of synaesthesia and stimuli. *Note*. Fa = false alarms; GC = Grapheme-colour synaesthetes; SC = Sound-colour synaesthetes; GCSC = Grapheme-and-Sound-colour synaesthetes; SS = Sequence-Space synaesthetes. Sound-colour- and sequence-space synaesthetes show a general advantage.
(DOCX)

S1 File Results including intelligence as covariate. (PDF)

S2 File DataFile. (SAV)
