# Supplementary material for: New insights into mechanisms of enhanced synaesthetic memory: Benefits are synaesthesia-type-specific
Source: PLoS One. 2018 Sep 5;13(9):e0203055. doi: 10.1371/journal.pone.0203055 (PMC6124748; doi:10.1371/journal.pone.0203055)
Supplement: S1 Table — Note. Fa = false alarms; GC = Grapheme-colour synaesthetes; SC = Sound-colour synaesthetes; GCSC = Grapheme-and-Sound-colour synaesthetes; SS = Sequence-Space synaesthetes. Sound-colour- and sequence-space synaesthetes show a general advantage. (DOCX) [file pone.0203055.s001.docx]

**Table 3. Proportions of hits and false alarms and C for each type of synaesthesia and stimuli.**

|  |  |  | GC | Controls | SC | Controls | GCSC | Controls | *SS* | Controls |
| --- | --- | --- | --- | --- | --- | --- | --- | --- | --- | --- |
|  |  |  | *M* (*SD*) | *M* (*SD*) | *M* (*SD*) | *M* (*SD*) | *M* (*SD*) | *M* (*SD*) | *M* (*SD*) | *M* (*SD*) |
| music | remember | hit | 0.38 (0.22) | 0.37 (0.16) | 0.46 (0.15) | 0.32 (0.17) | 0.45 (0.22) | 0.39 (0.21) | 0.40 (0.18) | 0.36 (0.17) |
|  |  | fa | 0.10 (0.11) | 0.12 (0.08) | 0.13 (0.11) | 0.09 (0.10) | 0.09 (0.09) | 0.10 (0.11) | 0.09 (0.09) | 0.11 (0.12) |
|  | know | hit | 0.34 (0.14) | 0.31 (0.15) | 0.23 (0.10) | 0.38 (0.15) | 0.28 (0.16) | 0.30 (0.17) | 0.33 (0.14) | 0.31 (0.14) |
|  |  | fa | 0.18 (0.11) | 0.20 (0.09) | 0.17 (0.12) | 0.23 (0.15) | 0.14 (0.08) | 0.21 (0.10) | 0.20 (0.09) | 0.18 (0.10) |
| words | remember | hit | 0.52 (0.25) | 0.61 (0.16) | 0.71 (0.19) | 0.66 (0.13) | 0.61 (0.25) | 0.60 (0.26) | 0.69 (0.17) | 0.65 (0.21) |
|  |  | fa | 0.08 (0.10) | 0.06 (0.06) | 0.06 (0.05) | 0.06 (0.10) | 0.07 (0.11) | 0.07 (0.09) | 0.04 (0.04) | 0.08 (0.11) |
|  | know | hit | 0.21 (0.13) | 0.15 (0.08) | 0.12 (0.08) | 0.17 (0.12) | 0.17 (0.13) | 0.17 (0.11) | 0.14 (0.08) | 0.13 (0.08) |
|  |  | fa | 0.16 (0.08) | 0.11 (0.08) | 0.11 (0.09) | 0.16 (0.13) | 0.11 (0.09) | 0.17 (0.17) | 0.15 (0.11) | 0.10 (0.13) |
| colours | remember | hit | 0.54 (0.17) | 0.49 (0.14) | 0.57 (0.17) | 0.44 (0.15) | 0.51 (0.17) | 0.53 (0.17) | 0.55 (0.15) | 0.51 (0.15) |
|  |  | fa | 0.08 (0.14) | 0.09 (0.10) | 0.09 (0.09) | 0.05 (0.06) | 0.09 (0.11) | 0.07 (0.08) | 0.07 (0.08) | 0.07 (0.07) |
|  | know | hit | 0.29 (0.13) | 0.31 (0.13) | 0.24 (0.14) | 0.35 (0.18) | 0.26 (0.15) | 0.30 (0.16) | 0.28 (0.13) | 0.27 (0.15) |
|  |  | fa | 0.17 (0.10) | 0.22 (0.14) | 0.18 (0.15) | 0.27 (0.13) | 0.18 (0.13) | 0.19 (0.13) | 0.19 (0.12) | 0.20 (0.14) |
| music | total | hit | 0.72 (0.15) | 0.68 (0.15) | 0.69 (0.14) | 0.70 (0.14) | 0.72 (0.15) | 0.69 (0.14) | 0.73 (0.14) | 0.67 (0.14) |
|  |  | fa | 0.27 (0.13) | 0.31 (0.10) | 0.29 (0.16) | 0.32 (0.20) | 0.23 (0.11) | 0.31 (0.12) | 0.29 (0.12) | 0.28 (0.11) |
| words | total | hit | 0.73 (0.20) | 0.76 (0.12) | 0.82 (0.14) | 0.83 (0.11) | 0.78 (0.15) | 0.78 (0.21) | 0.84 (0.11) | 0.78 (0.16) |
|  |  | fa | 0.24 (0.16) | 0.17 (0.12) | 0.17 (0.12) | 0.22 (0.19) | 0.18 (0.16) | 0.24 (0.22) | 0.18 (0.13) | 0.18 (0.17) |
| colours | total | hit | 0.83 (0.10) | 0.80 (0.11) | 0.81 (0.14) | 0.79 (0.16) | 0.78 (0.12) | 0.82 (0.12) | 0.83 (0.09) | 0.79 (0.13) |
|  |  | fa | 0.25 (0.15) | 0.30 (0.14) | 0.27 (0.14) | 0.32 (0.15) | 0.27 (0.14) | 0.26 (0.16) | 0.26 (0.13) | 0.27 (0.14) |
| music | C’ |  | -0.01 (0.79) | -0.01 (0.66) | 0.02 (0.74) | -0.10 (0.83) | 0.13 (0.67) | -0.07 (0.79) | -0.11 (0.59) | 0.14 (0.60) |
| words | C’ |  | 0.04 (0.87) | 0.17 (0.63) | -0.03 (0.58) | -0.20 (0.69) | 0.06 (0.73) | -.12 (1.13) | -0.12 (0.59) | 0.07 (0.86) |
| colours | C’ |  | -0.00 (0.83) | -0.09 (0.77) | -0.04 (0.90) | -0.11 (1.05) | 0.12 (0.79) | -0.04 (0.93) | -0.05 (0.66) | 0.10 (0.94) |

*Note.* Fa = false alarms; GC = Grapheme-colour synaesthetes; SC = Sound-colour synaesthetes; GCSC = Grapheme-and-Sound-colour synaesthetes; SS = Sequence-Space synaesthetes. Sound-colour- and sequence-space synaesthetes show a general advantage.
