# Supplementary material for: New insights into mechanisms of enhanced synaesthetic memory: Benefits are synaesthesia-type-specific
Source: PLoS One. 2018 Sep 5;13(9):e0203055. doi: 10.1371/journal.pone.0203055 (PMC6124748; doi:10.1371/journal.pone.0203055)
Supplement: S1 File — (PDF) [file pone.0203055.s002.pdf]

Results including intelligence as covariate

General memory performance. When verbal intelligence was included as covariate in the 2x4x3 repeated measures ANCOVA of recognition performance, the pattern of results did not change. A main effect occurred for *synaesthesia* (yes/no), $F(1, 184) = 4.20$, $MSE = 0.03$, $p = .042$, $\eta_p^2 = .02$, a two-way interaction occurred between *type of synaesthesia* and *type of stimuli*, $F(5.66, 347.42) = 2.29$, $MSE = 0.03$, $p = .038$, $\eta_p^2 = .04$ and a three-way interaction between *synaesthesia* (yes/no), *type of synaesthesia* and *type of stimuli*, $F(5.66, 347.42) = 3.19$, $MSE = 0.03$, $p = .006$, $\eta_p^2 = .05$. Intelligence itself showed a main effect, $F(1, 184) = 4.27$, $MSE = 0.03$, $p = .040$, $\eta_p^2 = .02$.

Following up on the three-way interaction, in the 2x3 repeated measures ANCOVAs the results were similar as without the covariate intelligence. For grapheme-colour synaesthetes occurred an interaction between *synaesthesia* (yes/no) and *type of stimuli*, $F(2, 100) = 6.70$, $MSE = 0.02$, $p = .002$, $\eta_p^2 = .12$, intelligence itself did not produce a main effect, $F(1, 50) = 2.12$, $MSE = 0.03$, $p = .152$, $\eta_p^2 = .04$. For grapheme-colour-and-sound-colour synaesthetes occurred the interaction between *synaesthesia* (yes/no) and *type of stimuli*, $F(1.71, 78.66) = 4.04$, $MSE = 0.03$, $p = .027$, $\eta_p^2 = .08$, and intelligence produced a main effect, $F(1, 46) = 4.12$, $MSE = 0.03$, $p = .048$, $\eta_p^2 = .08$.

Neither for C’ nor for recollection and familiarity did the inclusion of intelligence as a covariate change any of the relevant effects.
